# Bacille Calmette-Guérin Vaccine Strain Modulates the Ontogeny of Both Mycobacterial-Specific and Heterologous T Cell Immunity to Vaccination in Infants

**DOI:** 10.3389/fimmu.2019.02307

**Published:** 2019-10-01

**Authors:** Agano Kiravu, Sophia Osawe, Anna-Ursula Happel, Trishana Nundalall, Jerome Wendoh, Sophie Beer, Nobomi Dontsa, Olatogni Berenice Alinde, Sikiratu Mohammed, Pam Datong, D. William Cameron, Kenneth Rosenthal, Alash'le Abimiku, Heather B. Jaspan, Clive M. Gray

**Affiliations:** ^1^Division of Immunology, Institute of Infectious Diseases and Molecular Medicine, Department of Pathology, Faculty of Health Sciences, University of Cape Town, Cape Town, South Africa; ^2^Institute of Human Virology Nigeria, Abuja, Nigeria; ^3^Faculty of Biological Sciences, Friedrich Schiller University, Jena, Germany; ^4^Divisions of Infectious Diseases and Respirology, University of Ottawa at the Ottawa Hospital, Ottawa, ON, Canada; ^5^Department of Pathology and Molecular Medicine, McMaster University, Hamilton, ON, Canada; ^6^Institute of Human Virology, Department of Epidemiology and Prevention, University of Maryland School of Medicine, Baltimore, MD, United States; ^7^Department of Paediatrics and Global Health, University of Washington, Seattle, WA, United States; ^8^National Health Laboratory Services, Groote Schuur Hospital, Cape Town, South Africa

**Keywords:** BCG, vaccine strain, T cells, immunogenicity, Pertussis vaccine, Tetanus vaccine, ontogeny, Africa

## Abstract

Differences in Bacille Calmette-Guérin (BCG) immunogenicity and efficacy have been reported, but various strains of BCG are administered worldwide. Since BCG immunization may also provide protection against off-target antigens, we sought to identify the impact of different BCG strains on the ontogeny of vaccine-specific and heterologous vaccine immunogenicity in the first 9 months of life, utilizing two African birth cohorts. A total of 270 infants were studied: 84 from Jos, Nigeria (vaccinated with BCG-Bulgaria) and 187 from Cape Town, South Africa (154 vaccinated with BCG-Denmark and 33 with BCG-Russia). Infant whole blood was taken at birth, 7, 15, and 36 weeks and short-term stimulated (12 h) *in vitro* with BCG, Tetanus and Pertussis antigens. Using multiparameter flow cytometry, CD4+ T cell memory subset polyfunctionality was measured by analyzing permutations of TNF-α, IL-2, and IFN-γ expression at each time point. Data was analyzed using FlowJo, SPICE, R, and COMPASS. We found that infants vaccinated with BCG-Denmark mounted significantly higher frequencies of BCG-stimulated CD4+ T cell responses, peaking at week 7 after immunization, and possessed durable polyfunctional CD4+ T cells that were in a more early differentiated memory stage when compared with either BCG-Bulgaria and BCG-Russia strains. The latter responses had lower polyfunctional scores and tended to accumulate in a CD4+ T cell naïve-like state (CD45RA+CD27+). Notably, BCG-Denmark immunization resulted in higher magnitudes and polyfunctional cytokine responses to heterologous vaccine antigens (Tetanus and Pertussis). Collectively, our data show that BCG strain was the strongest determinant of both BCG-stimulated and heterologous vaccine stimulated T cell magnitude and polyfunctionality. These findings have implications for vaccine policy makers, manufacturers and programs worldwide and also suggest that BCG-Denmark, the first vaccine received in many African infants, has both specific and off-target effects in the first few months of life, which may provide an immune priming benefit to other EPI vaccines.

## Introduction

Many TB endemic countries provide Bacille Calmette-Guérin (BCG) vaccine to infants routinely soon after birth (1–4). BCG is efficacious against childhood TB, particularly extrapulmonary forms (5). Numerous strains of BCG exist, with various duplications and deletions in protein coding regions (6), which have been shown to affect the type of immunity elicited by the vaccine (1, 7, 8). Although no correlate of protection against *Mycobacterium tuberculosis* (mTB) exists (9), Th1 CD4+ T cells are believed to be important (10, 11) and are therefore used to measure BCG vaccine immunogenicity (1, 4, 7, 9, 12–14). BCG-Denmark strain has been shown to induce a greater magnitude and polyfunctional CD4+ Th1 cytokine response (8) and also has a range of heterologous effects (3, 15–17). For example, in adults, BCG-Denmark increases monocyte cytokine production against unrelated antigen stimulation; where such “trained immunity” after primary infection or vaccination has been shown to confer protection against secondary infection, independent of the adaptive immune system (16). Additionally, in adults BCG-Denmark increases Th1 and Th17 responses to *in vitro* stimulation with heterologous antigen (18). BCG-Denmark vaccinated infants had higher IFN-γ responses to Phytohaemagglutinin (PHA) and Tetanus Toxoid (TT) vs. those vaccinated with BCG-Russia in cultured whole blood supernatants (17). In low birth weight infants, BCG-Denmark vaccination has been associated with increased innate cytokine levels in whole blood stimulated with Toll-like receptor (TLR) ligands (19). Whether vaccine strains other than BCG-Denmark have a similar effect on T cell responses to unrelated antigens in newborn infants in Africa has not been assessed. In our study, we compared CD4+ T cell immunity to BCG, Tetanus and Pertussis vaccines in two cohorts of newborn infants recruited from Jos, Nigeria and Khayelitsha, Cape Town, South Africa, and then interrogated the effects of BCG strain further in the Cape Town cohort. We show that BCG vaccine strain not only impacts significantly on CD4+ T cell polyfunction and memory maturation, but also on heterologous T cell responses to other vaccines.

## Methods

### Cohort Description

Mother-infant pairs were recruited at the Midwifery Obstetric Unit (MOU) at Site B in Khayelitsha, Cape Town (CT), South Africa and the Plateau State Specialist Hospital in Jos, Nigeria from November 2014 to November 2016 (Table 1). Infants were followed from birth, at day 4–7 and at weeks 7, 15, and 36 of life. Voluntary counseling and HIV testing was done at the time of antenatal care registration. Both HIV-infected and HIV-uninfected mothers were eligible for the study. HIV-infected mothers and their infants were provided with anti-retrovirals (ARV) according to the current country-specific guidelines (20, 21). All mothers in the study were of consenting age and provided written informed consent. Exclusive breastfeeding (EBF) was advised to all mothers from delivery to at least 6 months. Infants born before 36 weeks and with birth weights lower than 2.4 kg were ineligible for the study. Further exclusion criteria included pregnancy or delivery complications as previously described (22). All HIV-exposed uninfected (HEU) infants were confirmed as negative by PCR at delivery and at later time points according to prevention of mother-to-child transmission (PMTCT) guidelines (20, 21).

**Table 1:** Infant participant characteristics.

	**BCG immunizing strain**			***P-*value**
	Denmark (*n* = 154)^*^	Russia (*n* = 32)^*^	**Bulgaria (*n* = 84)**	
Gestational age (weeks)	38 [37–39]	38 [38–39]	38 [38–39]	0.22
Birth weight (grams)	3,025 [2,778–3,330]	2,930 [2,840–3,230]	3,000 [2,700–3,300]	0.16
Birth length(centimeters)	48 [46–50]	47 [46–50]	47 [46–49]	0.10
Female (%)	89 (58)	18 (57)	34 (41)	0.29
HIV exposed (%)	105 (66)	17 (54)	65 (78)	0.03

* *Infants from Cape Town cohort*.

*Numbers represent median and [IQR]*.

*Kruskal-Wallis Test was applied to test differences across BCG strains for continuous variables*.

*Chi Squared test was used for categorical variables*.

### Immunization

Routine vaccines were given to all infants according to the WHO's Expanded Program on Immunization (EPI) (Supplementary Table 1). Infants from CT received intradermal Danish BCG strain (1331, Statens Serum Institute, Denmark) from April 2013 to January 2016 and thereafter Russian strain (BCG-I Moscow, Serum Institute of India, India). Both strains were given at 2 × 10^5^ CFU/dose at birth. Infants from Jos received the Bulgarian strain (BCG-SL 222 Sofia, BB-NCIPD Ltd., Bulgaria) at 0.3 × 10^5^ CFU/dose at 4–7 days after birth.

### Whole Blood Assay

A 12-h whole blood assay was used to evaluate vaccine responses as previously described (23). Briefly, 250 μL of anticoagulated blood was incubated with vaccine antigens or PHA at 37°C within 1 h of blood draw. For BCG, 12 × 10^5^ CFU/mL of *Mycobacterium bovis* was reconstituted in RPMI from either the vaccine vial (BCG-Denmark strain 1331; SSI or BCG-SL 222 Sofia strain; BB-NCIPD) or live BCG culture (BCG-Denmark strain 1331; AJVaccines, Denmark; Supplementary Table 2). TT antigen (Sanofi Pasteur) was used at 5 IU/mL and 0.01% v/v of Bordetella Pertussis (BP) antigens (BD Difco). PHA (50 μg/mL) or RPMI with 10% fetal calf serum (FCS) were used as positive and negative controls. After 5 h, Brefeldin-A (Sigma Aldrich) was added to a final concentration of 10 μg/mL and incubated for an additional 7 h. Thereafter, red blood cells were lysed followed by washing and staining with LIVE/DEAD® fixable Violet stain (ViViD, ThermoFisher). For the CT samples, cells were cryo-preserved in 10% DMSO and 90% FCS and stored immediately in liquid nitrogen (LN_2_). For Jos samples, cells were cryo-preserved in 10% DMSO and 90% FCS, stored at −70°C and shipped within 6 months to the University of Cape Town (UCT) and then transferred to LN_2_. Analysis of all samples was performed at the core laboratory at UCT.

### Cell Staining, Antibodies and Flow Cytometry

Batched stored samples were thawed quickly at 37°C and washed twice with 1X BD PermWash buffer. Cells were then incubated in 1X BD PermWash for 10 min before incubation with the antibody cocktail mix in 2% FCS at 4°C for 45 min. After incubation, cells were washed twice with PBS (2% FCS) and the resuspended in 0.3 mL PBS for cell acquisition using a Beckton Dickinson LSRII flow cytometer (SORF model). The following monoclonal antibody-fluorochrome conjugates were used: IL-2-R-phycoerythrin (PE), CD8-V500, IFN-γ-Alexa Fluor-700, TNF-α-PE-Cy7, Ki67-Fluorescein isothiocyanate (FITC), all from BD, CD27 PE-Cy5, HLA-DR- Allophycocyanin-Cy7 (APC-Cy7), CD3-BV650 (Biolegend), CD4 PE-Cy5.5 (Invitrogen), CD45RA PE-Texas Red-X (Beckman Coulter). A minimum of 50,000 ViViD negative (viable) CD3 events were collected using FACS DIVA v6 software. Post-acquisition compensation and analysis was performed in FlowJo version 9 (FlowJo, LLC). Supplementary Figure 1 shows the gating strategy employed.

### Statistical Analyses

All analysis was performed in R (24). Non-parametric comparisons between two independent groups was performed using Wilcoxon rank sum test (Mann Whitney *U*-test). Kruskal Wallis tests was used for longitudinal data. Holm's step down method was used for multiple comparison correction (25), and adjusted *P* values are reported. Cytokine combinations were assessed using SPICE software (26). For cytokine profiling analyses in SPICE, we only analyzed samples from participants classified as antigen responders (total CD4+ cytokine > 2 X median background) to avoid skewing related to samples that were likely to be background. In addition to SPICE, we used a more unbiased statistical approach by measuring polyfunctionality with COMPASS (Combinatorial polyfunctionality analysis of antigen- specific T-cell subsets) (27) to estimate the posterior probabilities of antigen specific T cell subsets. COMPASS is a statistical model for multi-functional (e.g., multiple cytokine subsets) analysis of flow cytometry data sets. Polyfunctionality scores which summarize the functional profile of each subject were calculated from posterior probabilities as described (27).

## Results

### Cohort Characteristics

A total of 186 samples from CT and 84 samples from Jos covering the first 9 months of life were included in this study (Table 1). There were no differences in gestational age at delivery, birth weight or length according to vaccine strain; BCG-Denmark, BCG-Bulgaria, and BCG-Russia. Although there were more infants who were born to HIV-infected mothers in the BCG-Bulgaria group, we identified no differences in any measured response between HIV-exposed and unexposed infants (Supplementary Table 3).

### Magnitude of Mycobacterial-Specific CD4+ T Cell Cytokine Responses Differ According to BCG Strain

**Figure 1:**
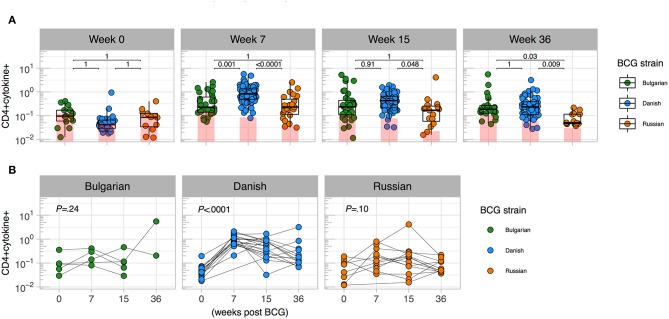
Magnitude of mycobacterial-specific CD4+ T cell cytokine responses differ between strains. **(A)** Cross-sectional analysis of mycobacterial-specific CD4 cytokine responses stratified by BCG immunizing strain. Weeks indicate the time point post vaccination with week 0 the pre-vaccination time point (birth for CT cohort and days 4–7 for Jos cohort). Y axes show the frequencies (%) of CD4+ cells producing total cytokine (any combination of IFN-γ, IL-2 or TNF-α) shown on a Log_10_ scale. Jitter point colors: Green (BCG-Bulgaria), blue (BCG-Denmark), orange (BCG-Russia), with shaded bars showing median value of unstimulated samples. Boxes with mid-line show interquartile ranges and median. Sample sizes by time point: week 0 (Bulgarian: 15, Danish: 35, Russian 14), week 7 (Bulgarian: 32, Danish: 83, Russian 35), week 15 (Bulgarian: 35, Danish: 66, Russian 17), week 36 (Bulgarian: 22, Danish: 60, Russian 17) Mann-Whitney *U* test was used to compare strains. Adjusted *P*-values are reported with *P* < 0.05 were considered significant after multiple comparisons correction. **(B)** Kinetics of BCG response stratified by BCG immunizing strain. Y axes show the frequencies (%) of CD4+ cells producing total cytokine (any combination of IFN-γ, IL-2 or TNF-α shown on a Log_10_ scale) for infants matched at least three time points between week 0 and week 36 (Bulgarian: 5, Danish: 21 Russian: 15). A Kruskal-Wallis test was applied to test differences by BCG strain across time (*P* < 0.05 were considered significant).

In January 2016, BCG-Denmark became unavailable and the South African EPI program began using BCG-Russia (Supplementary Table 1). BCG-Russia is genetically identical to BCG-Bulgaria (28), the strain given to infants in Nigeria. We hypothesized that immunogenicity to BCG-Denmark would differ to that of Russian and Bulgarian BCG immunized infants. We compared the magnitude of cytokine responses in infants vaccinated with both strains in CT vs. Jos. Infant samples were re-stimulated *in vitro* with the matched antigen, except cells from CT infants vaccinated with BCG-Russia were stimulated with BCG-Denmark culture (SSI) *in vitro* (Supplementary Table 2). We confirmed that the *in vitro* stimulating antigen did not impact the T cell cytokine magnitude and polyfunctional response (Supplementary Figure 2). As expected, total CD4 cytokine response magnitudes were comparable between strains before vaccination (Figures 1A,B). At week 7 post BCG vaccination, however, the BCG-Denmark strain vaccinated infants had significantly higher frequencies of CD4+ cytokine-producing cells over background (median = 0.83%) compared to both Jos infants vaccinated with BCG-Bulgaria (median = 0.23%) and CT infants vaccinated with BCG-Russia (median = 0.23%, *P* < 0.0001 for both; Figures 1A,B). Although there may be bias in the magnitude of responses in this cross-sectional analysis, our longitudinal data from matched samples across all time points emphasized that infants vaccinated with BCG-Denmark, peaking at week 7 (Figure 1B), had significantly higher CD4 responses compared to BCG-Russia vaccinated infants at weeks 7 (median = 0.92 vs. 0.11, *P* < 0.0001), 15 (median = 0.40 vs. 0.17, *P* = 0.03), and 36 (median = 0.23 vs. 0.05, *P* = 0.006; Figure 1B).

### Polyfunctional Mycobacterial-Specific CD4+ T Cell Cytokine Responses Differ Between Strains

**Figure 2:**
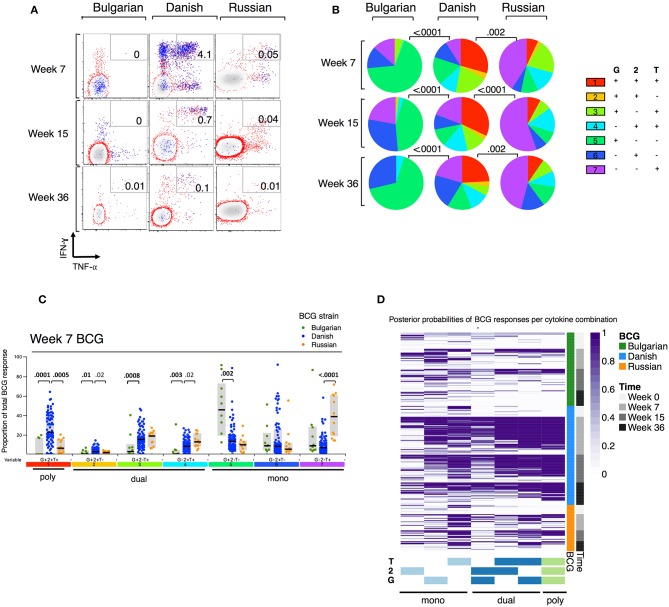
Polyfunction of mycobacterial-specific CD4+ T cell cytokine responses differ between strains. **(A)** Representative flow cytometry plots showing mycobacterial-specific cytokine expression by CD4+ cells stratified by BCG immunizing strain. Axes show IFN-γ vs. TNF-α expression with IL-2+ cells overlaid and represented by blue dots. Numbers indicate frequency of polyfunctional cells as a percentage of total CD4. **(B)** Pie charts show median proportions of cytokine combinations (G = IFN-γ, 2 = IL-2, T = TNF-α) as a fraction of the total response among antigen responders in each BCG strain group by SPICE analysis. Pie charts are compared using the SPICE permutations test with *P* < 0.05 considered significant. **(C)** Week 7 mycobacterial-specific cytokine profile by BCG immunizing strain. Jitter points show the median proportion of each cytokine combination (G = IFN-γ, 2 = IL-2, T = TNF-α) per infant as a fraction of total cytokine+ cells in infants who were responding to BCG and are color-coded by BCG immunizing strain. Shaded bars show interquartile ranges with line showing median. A Wilcoxon Rank Sum Test was used to compare cytokine combinations by strain with *P* < 0.01 considered significant after multiple comparison correction. **(D)** Heatmap of posterior probabilities of mycobacterial-specific responses estimated by COMPASS analysis. Rows represent individual infants and grouped by BCG immunizing strain and time post BCG vaccination (color-coded annotations are shown on right-hand side of heat map). Columns represent cytokine subsets (combinations) ordered by degree of functionality (single to polyfunctional; right to left). The color of each cell corresponds to the probability (range 0–1) of a mycobacterial-specific response for a particular cytokine cell subset per infant, with 1 indicating an antigen-specific response and white indicating background.

When we examined the combinations of IFN-γ and/or IL-2 and/or TNF-α-expressing CD4+ T cells by BCG strain, we observed that few polyfunctional cells were induced in infants receiving BCG-Bulgaria (Figures 2A,B), with the majority of responding CD4+ T cells being single positive for IFN-γ or IL-2 or TNF-α. Conversely, infants receiving BCG-Denmark had a high proportion of CD4+ T cells expressing all three cytokines, IFN-γ and IL-2 and TNF-α, at week 7. This was durable over time and constituted more than a quarter of the total cytokine response (Figures 2B,C). CT infants receiving BCG-Russia had significantly lower proportions of triple cytokine positive cells compared to BCG-Denmark (e.g, median at week 7, 7 vs. 38%, *P* = 0.001; Figure 2C). Furthermore, the proportion of dual-expressing IFN-γ+ and IL-2+ CD4+ T cells was higher among BCG-Denmark recipients compared to BCG-Russia recipients (3 vs. 2% at week 7, *P* = 0.02) and the proportions of dual-expressing IL-2+ and TNF-α+ cells was lower (median at week 7, 8 vs. 13%, *P* = 0.02). The majority of the total cytokine response among BCG-Russia recipients was made up of CD4+ T cells expressing single TNF-α; significantly more so than that induced by BCG-Denmark (median at week 7, 39 vs. 7%, respectively, *P* < 0.0001, Figure 2C). For the Bulgarian strain, the majority of responding CD4+ T cells were single IFN-γ, significantly more so than that induced by BCG-Denmark (median at week 7, 52 vs. 12%, respectively, *P* < 0.0001).

We evaluated polyfunctional scores (PFS) and posterior probabilities for each cytokine combination using COMPASS (27). As shown in the heatmap of posterior probabilities for all BCG responses in Figure 2D, there was a higher proportion of BCG-Denmark recipients with antigen-specific dual and polyfunctional responses compared with BCG-Russia and BCG-Bulgaria recipients. Only the BCG-Denmark strain elicited durable polyfunctional responses from 7 to 36 weeks as reflected in the PFS (Figure 2D, Supplementary Figure 3A). This more unbiased approach to Th1 polyfunctionality is compatible with our SPICE analyses (Figure 2B) and shows that BCG-Denmark elicits a more functional immune response in the newborn infant. Furthermore, these data highlight that the higher total cytokine expression is more likely to be a polyfunctional response, as observed through the significant association between PFS and the magnitude of BCG responses (Supplementary Figure 3B). Collectively, these data show that BCG-Denmark is more immunogenic than BCG-Bulgaria and BCG-Russia strains, characterized by a higher magnitude of response and a more polyfunctional cytokine profile. When only comparing CT infants i.e., BCG-Danish vs. BCG-Russia, thereby removing the potential confounding effect of cohort site, we found BCG-Denmark was significantly more immunogenic than BCG-Russia.

### BCG-Denmark Induces a More Differentiated T Cell Memory Phenotype Compared to BCG-Russia or BCG-Bulgaria Strains

**Figure 3:**
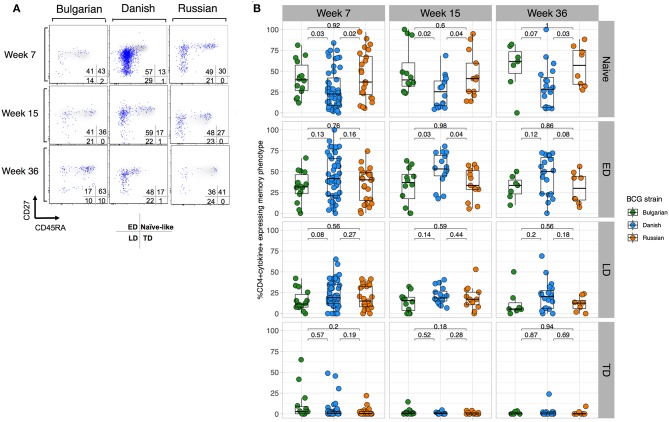
BCG-Denmark induces a more differentiated T cell memory phenotype compared to BCG-Russia or BCG-Bulgaria strains. **(A)** Representative flow cytometry plots showing the memory profile of mycobacterial-specific (cytokine positive) CD4 cytokine responses stratified by BCG immunizing strain. Axes show CD27 vs. CD45RA expression and blue dots are cytokine+ cells responding to BCG overlaid against a background of total CD4+ cells. CD45RA+CD27+ represent naïve-like, CD45RA–CD27+ early differentiated (ED), CD45RA–CD27– late differentiated (LD) and CD45RA+CD27– terminally differentiated (TD) phenotypes. **(B)** Frequency of mycobacterial-specific memory subsets stratified by BCG immunizing strain. Jitter point colors: Green (BCG-Bulgaria), blue (BCG-Denmark), orange (BCG-Russia), show the proportion of cytokine+ cells co-expressing combinations of memory markers CD45RA and CD27 that define memory subsets (as listed in **(A)**. Boxes with mid-line show interquartile ranges and median. Mann-Whitney *U* test was used to compare strains with *P* < 0.01 were considered significant after multiple comparisons correction.

To better understand whether BCG-Denmark may result in a more mature antigen-stimulated CD4+ T cell memory differentiation, which along with greater polyfunctionality provides important insight into protective responses against infections (29–32), we compared the memory phenotypes of the total cytokine BCG responding cells (i.e., cytokine positive) defined as naïve (CD27+CD45RA+), early differentiated (ED, CD27+CD45RA–), late differentiated (LD, CD27–CD45RA–) and terminally differentiated (TD, CD27–CD45RA+) CD4+ T cells. Our analysis of these subsets was restricted to only those infant cells that responded to BCG (>2-fold background). A large population of cytokine positive cells with a naïve phenotype (CD27+CD45RA+) was evident, which we hereon refer to as naïve-like (Figure 3A). Although there was a large range in the frequency of cytokine cells expressing memory subsets, we consistently observed that BCG-Denmark induced a lower frequency of these naïve-like cells across all time points post BCG vaccination (Figures 3A,B). BCG-Denmark induced a predominantly ED profile (Figure 3B), particularly at week 15 where BCG-Denmark induced the lowest frequencies of CD4+ cytokine positive cells expressing naïve-like markers (median = 22%) compared to BCG-Bulgaria and BCG-Russia (median = 40 and 36%, respectively, *P* = 0.02 and *P* = 0.04). Conversely, the CD4+ cytokine positive cells expressing ED markers were highest among BCG-Denmark vaccinated infants (median = 53%) compared to BCG-Bulgaria and BCG-Russia (37 and 33%; *P* = 0.03 and *P* = 0.04, respectively). Collectively, these data suggest that BCG-Denmark pushes cells into more differentiated memory state, which may explain the higher magnitude and polyfunctionality of response. Conversely, vaccination with BCG-Russia or -Bulgaria results in the probable accumulation of naïve-like CD4+ T cell responses.

### Strain of BCG Impacts CD4+ T Cell Responses to Heterologous Antigens

**Figure 4:**
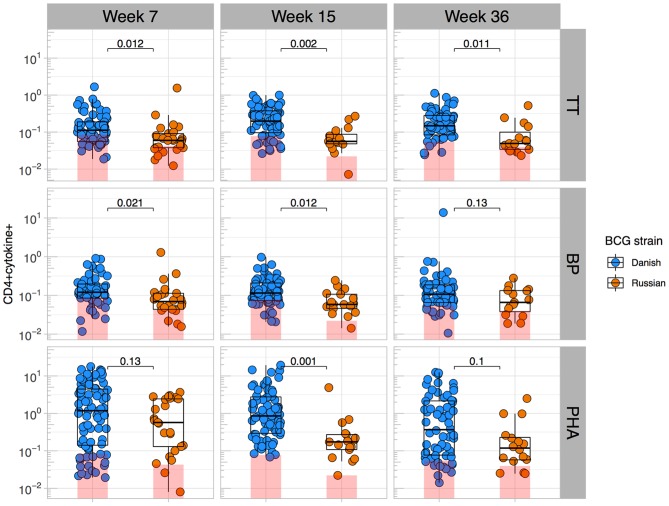
BCG strain impacts CD4+ T cells responses to heterologous antigens. Cross-sectional CD4 cytokine responses to heterologous antigens TT, BP, and PHA stratified by BCG immunizing strain. Y axes show the frequencies (%) of CD4+ cells producing total cytokine (any combination of IFN-γ, IL-2, or TNF-α) shown on a Log_10_ scale. Jitter point colors: blue (BCG-Denmark), orange (BCG-Russia), with shaded bars showing median value of unstimulated samples. Boxes with mid-line show interquartile ranges and median. Mann-Whitney *U* test was used to compare strains. Adjusted *P*-values are reported with *P* < 0.05 were considered significant after multiple comparisons correction.

**Figure 5:**
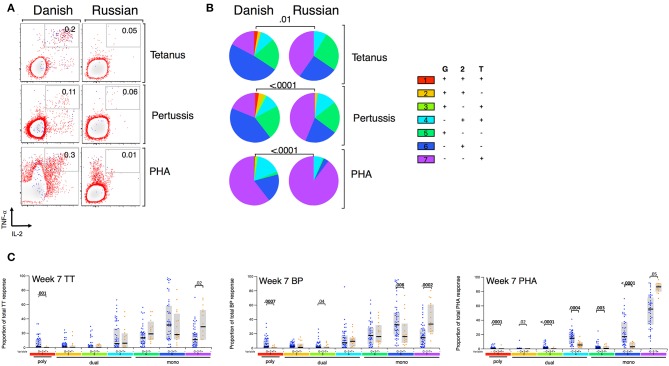
BCG strain impacts CD4+ T cells cytokine profile to heterologous antigens. **(A)** Representative flow cytometry plots showing TT, BP, and PHA-specific cytokine expression by CD4+ cells stratified by BCG immunizing strain at week 7. Numbers indicate frequency of polyfunctional cells as a percentage of total CD4. Axes show IFN-γ vs. TNF-α expression with IL-2+ cells overlaid and represented by blue dots. **(B)** Pie charts show median proportions of cytokine combinations (G = IFN-γ, 2 = IL-2, T = TNF-α) as a fraction of the total response among antigen responders in each BCG strain group by SPICE analysis. Pie charts are compared using the SPICE permutations test with *P* < 0.05 considered significant. **(C)** Week 7 heterologous antigens TT, BP, and PHA cytokine profile by BCG immunizing strain. Jitter points show the median proportion of each cytokine combination per infant as a fraction of total cytokine+ cells in infants who were responding to antigen and are color-coded by BCG immunizing strain [Green (BCG-Bulgaria), blue (BCG-Denmark), orange (BCG-Russia)]. Shaded bars show interquartile ranges with line showing median. A Wilcoxon Rank Sum Test was used to compare cytokine combinations by strain with *P* < 0.01 considered significant after multiple comparison correction.

Given that BCG has been shown to have heterologous effects to non-BCG antigens (3, 16, 17, 33), we examined the effect of BCG strain on CD4+ T cell responses to TT, BP, and PHA. We focused only on CT infants and compared those who received BCG-Denmark vs. BCG-Russia strains, and thus removed the potential confounding effects of geography, genetic background of participants and different vaccination strategies. Infants vaccinated with BCG-Denmark had significantly higher frequencies of total Th1 cytokine-producing cells to TT, BP, and PHA compared to those had receiving BCG-Russia (Figure 4). This was most marked at week 15 after birth where, on average, infants vaccinated with BCG-Denmark mounted 3-fold higher responses to TT over BCG-Russia (Figure 4, median = 0.2 vs. 0.06%, respectively, *P* = 0.002), 2-fold for BP response (median = 0.12 vs. 0.06%, respectively, *P* = 0.01) and 5-fold PHA response (median 0.9 vs. 0.17% respectively, *P* = 0.001). These BCG-strain-related effects were also evident at week 7 for BP and TT antigens, despite the fact that the first dose of these vaccines is given at 6 weeks of age (Supplementary Table 1). We examined the polyfunctionality of these responses at week 7, when the BCG response itself peaked (Figures 5A–C). The BCG-Denmark strain also induced significantly higher proportions of polyfunctional (IFN-γ+ and TNF-α+ and IL-2+) CD4 cells to TT (*P* = 0.001), BP (*P* = 0.0007), and PHA (*P* = 0.0001) (Figure 5C). There was also a significantly higher proportion of single TNF-α+ responding CD4+ T cells to TT, BP, and PHA (Figure 5C) after BCG-Russia immunization. Using COMPASS analysis for TT and BP responses, we found that BCG-Denmark was associated with higher probabilities of polyfunctional and dual functional antigen-specific responses (Supplementary Figure 4). Specifically, BCG-Denmark induced higher PFS to TT at week 36 (*P* = 0.01) and a slight trend towards higher PFS to BP at week 36. Collectively, our data show that the strain used for BCG vaccination has a profound impact on the magnitude and polyfunctionality of CD4+ T cell responses to unrelated heterologous vaccine antigens.

## Discussion

Our study shows that the magnitude and polyfunctional nature of CD4+ T cell responses to BCG and other heterologous antigens in the first few months of life depends on the immunizing strain of BCG itself. BCG-Denmark is the most immunogenic compared with BCG-Russia and BCG-Bulgaria and causes an early differentiated phenotype in memory CD4+ T cells. The other strains appear to allow the accumulation of naïve-like responsive cells, which seem to be more mono-functional. The important aspect of our study is that responding CD4+ T cells to all vaccine antigens, including Tetanus and Pertussis, were significantly affected by the strain of immunizing BCG, with the Denmark strain inducing the highest magnitude of responses and a Th1 polyfunctional subset. Furthermore, the relatively advanced stage of CD4+ T cell memory maturation in response to BCG-Denmark may explain the phenomena of infant cells responding with greater magnitude and Th1 polyfunction.

Prior studies have suggested that BCG-Denmark may be more immunogenic than other widely available BCG strains. A randomized control trial in Uganda administering the same BCG strains used in this study showed that BCG-Denmark induced the highest levels of IFN-γ, IL-13, and IL-5 in culture supernatants to BCG, TT, and PHA stimulation (17). The cellular source of cytokine production, however, was not measured due to the nature of the assay used. In support of our data, CD4 polyfunctionality among infants randomized to receive BCG-Denmark, -Japan and -Russia at birth showed higher polyfunctional (IFN-γ+TNF-α+IL-2+) responses after BCG-Denmark strain immunization (7). Our findings are similar, except that our cohorts are in some of the highest TB endemic areas in Africa (13, 34–36) and that the early differentiated memory state may also be important for the heterologous non-BCG antigen effects we observe with BCG-Denmark. Although CD4+ Th1 cytokine responses are not cognate correlates of protection against Tetanus and Pertussis, they are likely important measures of overall immune responsiveness to heterologous antigens.

The attenuation of BCG is the result of the loss of virulent elements in the genome, with some strains having more regions of deletions than others (6, 37, 38). BCG-Denmark, a member of the DU2-III group, has more deletions than both BCG-Russia and BCG-Bulgaria from group DU2-I (6). The better quality and quantity of responses induced by BCG-Denmark are unlikely-related to antigenic coverage as this strain has less T cell epitopes than BCG-Russia (38). Certain BCG strains are known to replicate and persist longer in tissues after immunization in animal models (39, 40) and this may result in different antigen priming between strains. However, there are most likely numerous factors other than antigen load, as BCG-Russia has been found to replicate and persist in tissues of immunized mice over and above BCG-Denmark related strains (BCG-Prague and BCG-Glaxo strains) (40).

There are likely numerous possible reasons for differences in vaccine immunogenicity between CT and Jos. We do not regard the 6-fold lower CFU dose of BCG-Bulgaria, compared with BCG-Denmark/Russia, as a contributing factor (Supplementary Table 2). Suboptimal vaccination doses affect the magnitudes of Th1 responses in adults (41), but not infants (42). Differences in immune responses between cohorts may also depend on population differences; i.e., environmental antigen exposure and/or genetics (43–45). For example helminth and malaria co-infections, which are common in Nigeria, are associated with IL-10 responses and a skewing to Th2 response to mTB (46), but we did not evaluate helminth infections in our infant cohort. Shipping of cells from Nigeria to South Africa may have impacted on their integrity. However, our finding that BCG-Russia [which is closely related to BCG-Bulgaria (28)] also resulted in a similar set of low magnitude and polyfunctional responses in CT infants led us to regard BCG strain differences as a main determinant of vaccine immunogenicity. Finally, although the proportion of HIV-exposed infants was unbalanced between the groups, this would have favored better responses in the BCG-Bulgarian infants.

In 2016, many countries around the world experienced a shortage of BCG-Denmark and had to transition to other strains (47). Our results suggest that BCG vaccination, given to over 100 million infants annually around the globe, has variable immunogenicity according to strain, and that heterologous effects may be specific to BCG-Denmark. These findings have implications for vaccine policy makers, manufacturers and programs worldwide. These findings also suggest that BCG-Denmark, the first vaccine received in many African infants, has both specific and non-specific heterologous effects in the first few months of life, and may provide an immune priming benefit to other EPI vaccines.

## Data Availability Statement

All datasets generated for this study are included in the manuscript/Supplementary Files.

## Ethics Statement

The studies involving human participants were reviewed and approved by University of Cape Town Human Research Ethics Committee. Written informed consent to participate in this study was provided by the participants' legal guardian/next of kin.

## Author Contributions

DC, KR, AA, HJ, and CG designed the study. AK, HJ, and CG interpreted that data and wrote the manuscript. AK, SO, TN, JW, SB, ND, OA, and SM ran the laboratory experiments. AK, AH, and HJ analyzed the data. SO, PD, AA, and HJ oversaw the recruitment and follow-up of mother-infant pairs.

### Conflict of Interest

The authors declare that the research was conducted in the absence of any commercial or financial relationships that could be construed as a potential conflict of interest.

## References

[1] Davids V Hanekom WA Mansoor N Gamieldien H Gelderbloem SJ Hawkridge A. The effect of bacille Calmette-Guérin vaccine strain and route of administration on induced immune responses in vaccinated infants. J Infect Dis. (2006) 193:531–6. 10.1086/499825 16425132

[2] Randhawa AK Shey MS Keyser A Peixoto B Wells RD de Kock M. Association of human TLR1 and TLR6 deficiency with altered immune responses to bcg vaccination in south african infants. PLoS Pathog. (2011) 7:e1002174. 10.1371/journal.ppat.1002174 21852947 PMC3154845

[3] Frankel H Byberg S Bjerregaard-Andersen M Martins CL Aaby P Benn CS. Different effects of BCG strains – a natural experiment evaluating the impact of the Danish and the Russian BCG strains on morbidity and scar formation in Guinea-Bissau. Vaccine. (2016) 34:4586–93. 10.1016/j.vaccine.2016.07.022 27491688

[4] Kidzeru EB Hesseling AC Passmore JAS Myer L Gamieldien H Tchakoute CT. *In-utero* exposure to maternal HIV infection alters T-cell immune responses to vaccination in HIV-uninfected infants. AIDS. (2014) 28:1421–30. 10.1097/QAD.0000000000000292 24785950 PMC4333196

[5] Pitt JM Blankley S McShane H O'Garra A. Vaccination against tuberculosis: how can we better BCG? Microb Pathog. (2013) 58:2–16. 10.1016/j.micpath.2012.12.002 23257069

[6] Mostowy S Tsolaki AG Small PM Behr MA. The *in vitro* evolution of BCG vaccines. Vaccine. (2003) 21:4270–4. 10.1016/S0264-410X(03)00484-5 14505909

[7] Ritz N Dutta B Donath S Casalaz D Connell TG Tebruegge M. The influence of bacille Calmette-Guérin vaccine strain on the immune response against tuberculosis: a randomized trial. Am J Respir Crit Care Med. (2012) 185:213–22. 10.1164/rccm.201104-0714OC 22071384

[8] Ritz N Hanekom WA Robins-Browne R Britton WJ Curtis N. Influence of BCG vaccine strain on the immune response and protection against tuberculosis. FEMS Microbiol Rev. (2008) 32:821–41. 10.1111/j.1574-6976.2008.00118.x 18616602

[9] Kagina BMN Abel B Scriba TJ Hughes EJ Keyser A Soares A Specific T cell frequency and cytokine expression profile do not correlate with protection against tuberculosis after bacillus Calmette-Guérin vaccination of newborns. Am J Respir Crit Care Med. (2010) 182:1073–9. 10.1164/rccm.201003-0334OC 20558627 PMC2970848

[10] Cooper BAM Dalton DK Stewart TA Griffin JP Russell DG Orme IM Disseminated Tuberculosis in Interferon 7 Gene-disrupted Mice. J Exp Med. (1993) 178:2243–7. 10.1084/jem.178.6.2243 8245795 PMC2191280

[11] Flynn JL. An essential role for interferon gamma in resistance to *Mycobacterium tuberculosis* infection. J Exp Med. (2004) 178:2249–54. 10.1084/jem.178.6.2249 7504064 PMC2191274

[12] Mansoor N Scriba TJ Kock M De Tameris M Keyser A Little F Infant HIV-1 infection severely impairs the bacille Calmette-Guérin vaccine-induced immune response. J Infect Dis. (2010) 199:1–17. 10.1086/597304 PMC2815505 19236280

[13] Tchakoute CT Hesseling AC Kidzeru EB Gamieldien H Passmore JAS Jones CE. Delaying BCG vaccination until 8 weeks of age results in robust BCG-specific T-cell responses in HIV-exposed infants. J Infect Dis. (2015) 211:338–46. 10.1093/infdis/jiu434 25108027 PMC4318913

[14] Garcia-Knight MA Nduati E Hassan AS Gambo F Odera D Etyang TJ. Altered memory T-cell responses to Bacillus Calmette-Guerin and Tetanus Toxoid vaccination and altered cytokine responses to polyclonal stimulation in HIV-exposed uninfected Kenyan infants. PLoS ONE. (2015) 10:1–19. 10.1371/journal.pone.0143043 26569505 PMC4646342

[15] Rentsch CA Birkhäuser FD Biot C Gsponer JR Bisiaux A Wetterauer C. Bacillus calmette-guérin strain differences have an impact on clinical outcome in bladder cancer immunotherapy. Eur Urol. (2014) 66:677–88. 10.1016/j.eururo.2014.02.061 24674149

[16] Kleinnijenhuis J Quintin J Preijers F Joosten LAB Ifrim DC Saeed S. Bacille Calmette-Guerin induces NOD2-dependent nonspecific protection from reinfection via epigenetic reprogramming of monocytes. Proc Natl Acad Sci USA. (2012) 109:17537–42. 10.1073/pnas.1202870109 22988082 PMC3491454

[17] Anderson EJ Webb EL Mawa PA Kizza M Lyadda N Nampijja M. The influence of BCG vaccine strain on mycobacteria-specific and non-specific immune responses in a prospective cohort of infants in Uganda. Vaccine. (2012) 30:2083–9. 10.1016/j.vaccine.2012.01.053 22300718 PMC3314967

[18] Kleinnijenhuis J Quintin J Preijers F Benn CS Joosten LAB Jacobs C. Long-lasting effects of BCG vaccination on both heterologous Th1/Th17 responses and innate trained immunity. J Innate Immun. (2014) 6:152–8. 10.1159/000355628 24192057 PMC3944069

[19] Jensen KJ Larsen N Sørensen SB Andersen A Eriksen HB Monteiro I. Heterologous immunological effects of early BCG vaccination in low-birth-weight infants in guinea-bissau: a randomized-controlled trial. J Infect Dis. (2015) 211:956–67. 10.1093/infdis/jiu508 25210141 PMC4340366

[20] National Department of Health South Africa 2015 National Consolidated Guidelines. (2015). p. 1–78.

[21] Federal Ministry of Health National AIDS and STI's Control Programme, Federal Ministry of Health: National Guidelines for HIV Prevention Treatment and Care (2016). (2016). Available online at: http://apps.who.int/medicinedocs/documents/s23252en/s23252en.pdf

[22] Tchakoute CT Sainani KL Osawe S Datong P Kiravu A Rosenthal KL. Breastfeeding mitigates the effects of maternal HIV on infant infectious morbidity in the Option B+ era. AIDS. (2018) 32:2383–91. 10.1097/QAD.0000000000001974 30134300

[23] Hanekom WA Hughes J Mavinkurve M Mendillo M Watkins M Gamieldien H. Novel application of a whole blood intracellular cytokine detection assay to quantitate specific T-cell frequency in field studies. J Immunol Methods. (2004) 291:185–95. 10.1016/j.jim.2004.06.010 15345316

[24] R Core Team. R: A Language and Environment for Statistical Computing. Vienna: R Foundation for Statistical Computing (2014). Available online at: http://www.R-project.org/

[25] Holm S Board of the Foundation of the Scandinavian Journal of Statistics. A simple sequentially rejective multiple test procedure. Source Scand J Stat. (1979) 6:65–70.

[26] Roederer M Nozzi JL Nason MC SPICE: exploration and analysis of post-cytometric complex multivariate datasets. Cytom Part A. (2011) 79A:167–74. 10.1002/cyto.a.21015 PMC3072288 21265010

[27] Lin L Finak G Ushey K Seshadri C Hawn TR Frahm N. COMPASS identifies T-cell subsets correlated with clinical outcomes. Nat Biotechnol. (2015) 33:610–6. 10.1038/nbt.3187 26006008 PMC4569006

[28] Stefanova T Chouchkova M Hinds J Butcher PD Inwald J Dale J. genetic composition of *Mycobacterium bovis* BCG substrain sofia [2]. J Clin Microbiol. (2003) 41:5349. 10.1128/JCM.41.11.5349.2003 14605203 PMC262522

[29] Darrah PA Patel DT De Luca PM Lindsay RWB Davey DF Flynn BJ Multifunctional T H 1 cells define a correlate of vaccine- mediated protection against Leishmania major. Nat Med. (2007) 13:843–50. 10.1038/nm1592 17558415

[30] Kannanganat S Kapogiannis BG Ibegbu C Chennareddi L Goepfert P Robinson HL Human immunodeficiency virus Type 1 controllers but not noncontrollers maintain CD4 T cells coexpressing three cytokines. J Virol. (2007) 81:12071–76. 10.1128/JVI.01261-07 17728221 PMC2168799

[31] Bhatt K Verma S Ellner JJ Salgame P. Quest for correlates of protection against tuberculosis. Clin Vaccine Immunol. (2015) 22:258–66. 10.1128/CVI.00721-14 25589549 PMC4340894

[32] Seder RA Darrah PA Roederer M. T-cell quality in memory and protection: implications for vaccine design. Nat Rev Immunol. (2008) 8:247–58. 10.1038/nri2274 18323851

[33] Kleinnijenhuis J Van Crevel R Netea MG. Trained immunity: consequences for the heterologous effects of BCG vaccination. Trans R Soc Trop Med Hyg. (2014) 109:29–35. 10.1093/trstmh/tru168 25573107

[34] Jones CE Hesseling AC Tena-Coki NG Scriba TJ Chegou NN Kidd M. The impact of HIV exposure and maternal *Mycobacterium tuberculosis* infection on infant immune responses to bacille Calmette-Guérin vaccination. AIDS. (2015) 29:155–65. 10.1097/QAD.0000000000000536 25535752 PMC4284011

[35] Soares AP Scriba TJ Joseph S Harbacheuski R Murray RA Gelderbloem SJ. Bacillus calmette-guerin vaccination of human newborns induces T cells with complex cytokine and phenotypic profiles. J Immunol. (2014) 180:3569–77. 10.4049/jimmunol.180.5.3569 18292584 PMC2842001

[36] Cranmer LM Draper HR Mandalakas AM Kim S McSherry G Krezinski E. High incidence of tuberculosis infection in HIV-exposed children exiting an isoniazid preventive therapy trial. Pediatr Infect Dis J. (2018) 37:e254–6. 10.1097/INF.0000000000001946 29462104 PMC6095832

[37] Behr MA Wilson MA Gill WP Salamon H Schoolnik GK Rane S. Comparative genomics of BCG vaccines by whole-genome DNA microarray. Science. (1999) 284:1520–3. 10.1126/science.284.5419.1520 10348738

[38] Zhang W Zhang Y Zheng H Pan Y Liu H Du P. Genome sequencing and analysis of BCG vaccine strains. PLoS ONE. (2013) 8:1–7. 10.1371/journal.pone.0071243 23977002 PMC3747166

[39] Zhang L Ru HW Chen FZ Jin CY Sun RF Fan XY. Variable virulence and efficacy of BCG vaccine strains in mice and correlation with genome polymorphisms. Mol Ther. (2016) 24:398–405. 10.1038/mt.2015.216 26643797 PMC4817822

[40] Lagranderie MR Balazuc a M Deriaud E Leclerc CD Gheorghiu M. Comparison of immune responses of mice immunized with five different *Mycobacterium bovis* BCG vaccine strains. Infect Immun. (1996) 64:1–9. 8557324 10.1128/iai.64.1.1-9.1996 PMC173719

[41] Lowry PW Ludwig TS Adams JA Fitzpatrick ML Grant SM Andrle GA. Cellular immune responses to four doses of percutaneous Bacille Calmette-Guerin in healthy adults. J Infect Dis. (2012) 178:138–46. 10.1086/515614 9652433

[42] Davids V Hanekom W Gelderbloem SJ Hawkridge A Hussey G Sheperd R. Dose-dependent immune response to *Mycobacterium bovis* BCG vaccination in neonates. Clin Vaccine Immunol. (2007) 14:198–200. 10.1128/CVI.00309-06 17182761 PMC1797790

[43] Lalor MK Ben-Smith A Gorak-Stolinska P Weir RE Floyd S Blitz R. Population differences in immune responses to Bacille Calmette-Guérin vaccination in infancy. J Infect Dis. (2009) 199:795–800. 10.1086/597069 19434928 PMC3276835

[44] van den Biggelaar AHJ Prescott SL Roponen M Nadal-Sims MA Devitt CJ Phuanukoonnon S. Neonatal innate cytokine responses to BCG controlling T-cell development vary between populations. J Allergy Clin Immunol. (2009) 124:544–50 e2. 10.1016/j.jaci.2009.03.040 19500827

[45] Elias D Britton S Aseffa A Engers H Akuffo H. Poor immunogenicity of BCG in helminth infected population is associated with increased *in vitro* TGF-β production. Vaccine. (2008) 26:3897–902. 10.1016/j.vaccine.2008.04.083 18554755

[46] Elliott AM Mawa PA Webb EL Nampijja M Lyadda N Bukusuba J. Effects of maternal and infant co-infections, and of maternal immunisation, on the infant response to BCG and tetanus immunisation. Vaccine. (2010) 29:247–55. 10.1016/j.vaccine.2010.10.047 21040693 PMC3021124

[47] Cernuschi T Malvolti S Nickels E Friede M. Bacillus Calmette-Guérin (BCG) vaccine: a global assessment of demand and supply balance. Vaccine. (2018) 36:498–506. 10.1016/j.vaccine.2017.12.010 29254839 PMC5777639

