# Supplementary material for: Bacille Calmette-Guérin Vaccine Strain Modulates the Ontogeny of Both Mycobacterial-Specific and Heterologous T Cell Immunity to Vaccination in Infants
Source: Front Immunol. 2019 Oct 1;10:2307. doi: 10.3389/fimmu.2019.02307 (PMC6793433; doi:10.3389/fimmu.2019.02307)
Supplement: Supplementary file 1 [file Data_Sheet_2.docx]

**SUPPLEMENTARY TABLE 1** | Infant vaccine schedule and whole blood assay testing

|  | **Vaccines** | | **WBA testing** | | |
| --- | --- | --- | --- | --- | --- |
| **Time point** | South Africa | Nigeria | South Africa | | Nigeria |
| Birth | BCG**^#^**, OPV, HBV |  | X | |  |
| Days 4-7 |  | BCG, OPV, HBV, PCV | | X | X |
| Week 6 | DTaP, Hib, OPV, HBV, RV, PCV | DTwP, Hib, OPV, HBV | |  |  |
| Week 7 |  |  | | X | X |
| Week 10 | DTaP, Hib, HBV | DTwP, Hib, HBV, OPV | |  |  |
| Week 14 | DTaP, Hib, Rota, PCV, HBV | DTwP, Hib, OPV, PCV | |  |  |
| Week 15 |  |  | | X | X |
| Week 36 |  |  | | X | X |

*Vaccines: BCG- Bacille-Calmette Guerin,* ***^#^****BCG-Denmark* *prior to Jan 2016 and BCG-Russia thereafter. DTaP-Diptheria-Tetanus-acellular Pertusiss, DTP- DTwP-Diptheria-Tetanus-Whole-cell-Pertusiss, OPV-Oral Polio Vaccine, Hib- Haemophilus influenzae type b, HBV- Hepatitis B Vaccine PCV- Pneumococcal conjugate vaccine.*

*X- WBA testing time points.*

**SUPPLEMENTARY TABLE 2** | In vivo and in vitro vaccine antigen concentrations

| **Cohort** | **Vaccine antigen dose** | ***In vitro* antigen concentration** |
| --- | --- | --- |
| **CT** | Denmark strain 1331 (~2 x 10^5^ CFU) | Denmark strain 1331(~ 12 x 10^5^ CFU/ml) |
|  | Tetanus Toxoid (>40 IU) | Tetanus Toxoid antigen (5 IU/ml) |
|  | Acellular Pertussis (25 micrograms) | Bordetella antigens (0.1%v/v) |
| **Jos** | Bulgaria strain (SL 222 Sofia) (~0.3 x 10^5^ CFU) | Bulgaria strain (SL 222 Sofia) (144 CFU/ml) |
|  | Tetanus Toxoid (>40 IU) | Tetanus Toxoid antigen (5 IU/ml) |
|  | Whole cell Pertussis (>4.0IU) | Bordetella antigens (0.1%v/v) |

Numbers represent approximate dose per infant and approximate in vitro antigen concentration per ml of whole blood.

**SUPPLEMENTARY TABLE 3** | Magnitude of infant mycobacterial-specific CD4 cytokine responses by maternal HIV status

| **Time (N)** | **Median CD4 cyk & [IQR]** | |  |
| --- | --- | --- | --- |
|  | HEU | HU | *P*-values |
| Week 0 (28 vs. 7) | 0.04 [0.03-0.06] | 0.04 [0.03-0.08] | 0.98 |
| Week 7 (58 vs. 27) | 0.77 [0.48-1.46] | 1.01 [0.40-1.28] | 0.97 |
| Week 15 (42 vs. 24) | 0.47 [0.22-0.65] | 0.41 [0.20-0.70] | 0.53 |
| Week 36 (43 vs. 21) | 0.20 [0.09-0.37] | 0.22 [0.16-0.52] | 0.19 |

A comparison of CD4 responses among the BCG Denmark vaccinated group between infants born to HIV+ mothers (defined as HIV exposed and uninfected: HEU) and HIV- mothers (defined as HIV unexposed: HU). Time point show the weeks post BCG vaccination with brackets showing the numbers compared (HEU/HU). Values indicate median frequencies of CD4+ cells producing total cytokine (any combination of IFN-g, IL-2 or TNF-a) with interquartile ranges in square brackets.: A Wilcoxon Rank Sum Test was used to compare infant responses by maternal HIV status.
