# Supplementary material for: Bacille Calmette-Guérin Vaccine Strain Modulates the Ontogeny of Both Mycobacterial-Specific and Heterologous T Cell Immunity to Vaccination in Infants
Source: Front Immunol. 2019 Oct 1;10:2307. doi: 10.3389/fimmu.2019.02307 (PMC6793433; doi:10.3389/fimmu.2019.02307)
Supplement: Supplementary file 2 [file Data_Sheet_1.pdf]

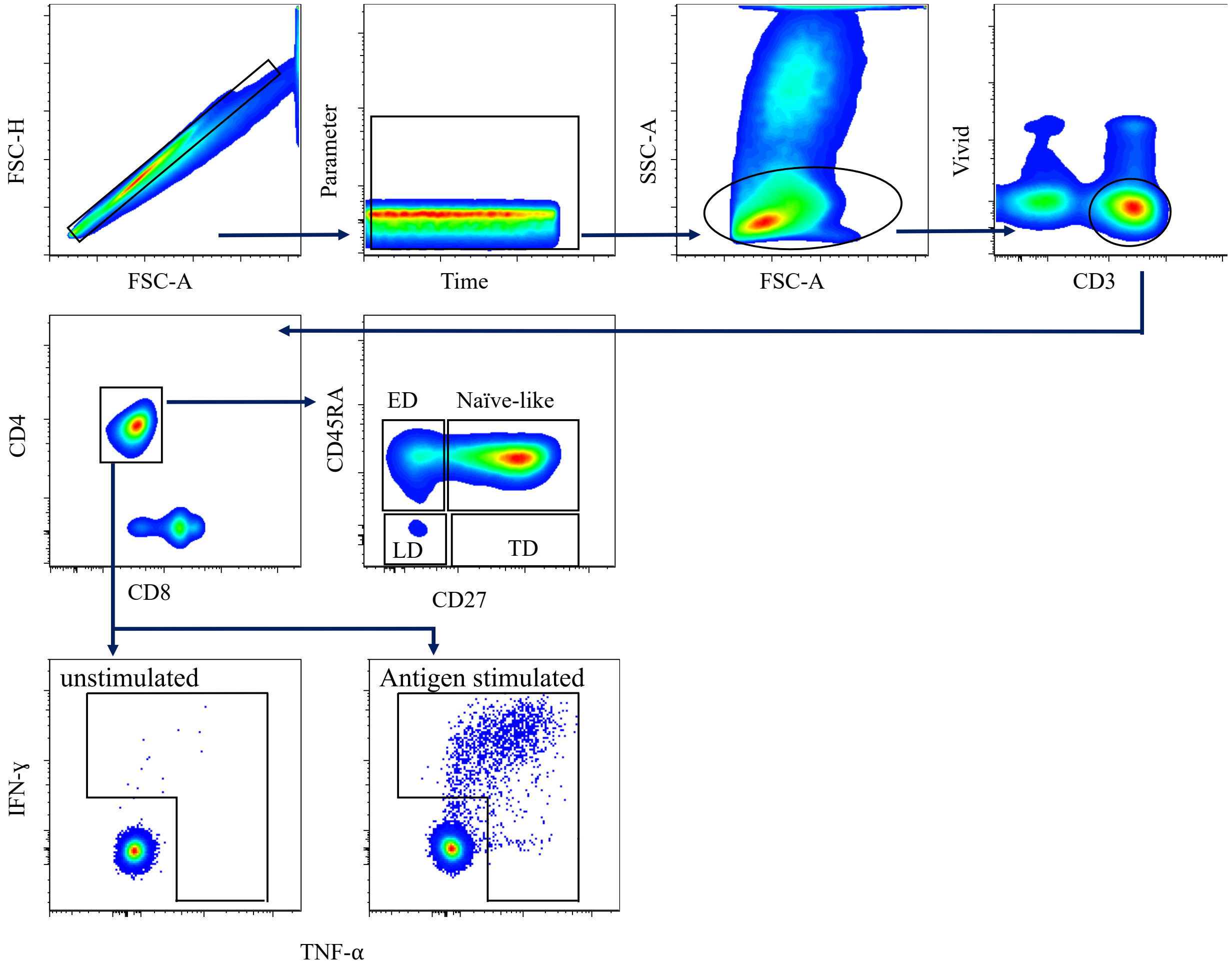

Supplementary 1 Flow cytometry gating strategy for quantifying antigen specific responses. Cytokine responses were measured from the parent CD4+ population. Cytokine memory subsets were quantified by applying memory gates from parent CD4+ population to Boolean-defined total cytokine population in antigen stimulated samples. CD45RA+CD27+ represent naïve-like, CD45RA-CD27+ early differentiated (ED), CD45RA-CD27- late differentiated (LD) and CD45RA+CD27- terminally differentiated (TD) memory phenotypes.

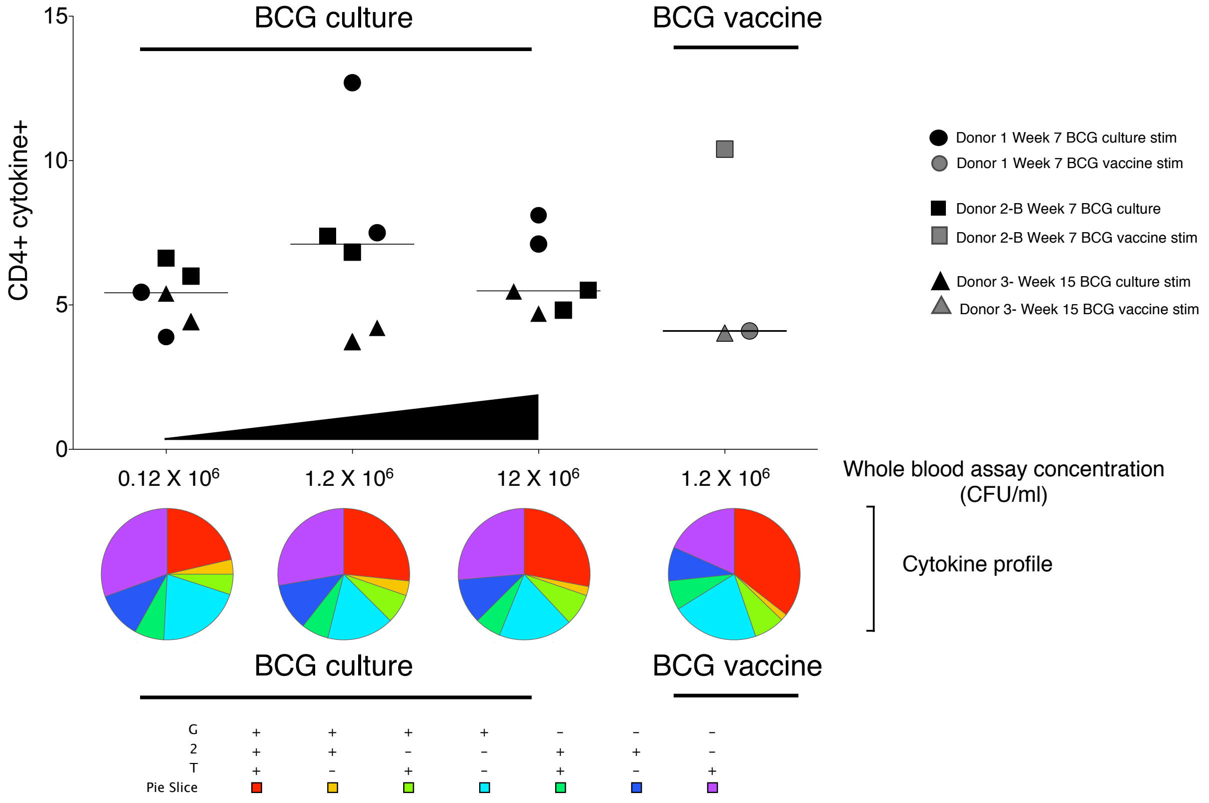

Supplementary 2 BCG vaccine vs. BCG culture re-stimulation

Comparison of different *in vitro* concentrations of BCG culture (Danish SSI strain) compared to the standard BCG vaccine *in vitro* concentration on CD4 response magnitudes and polyfunction. Each point is represents an infant replicate under different *in vitro* stimulation conditions.

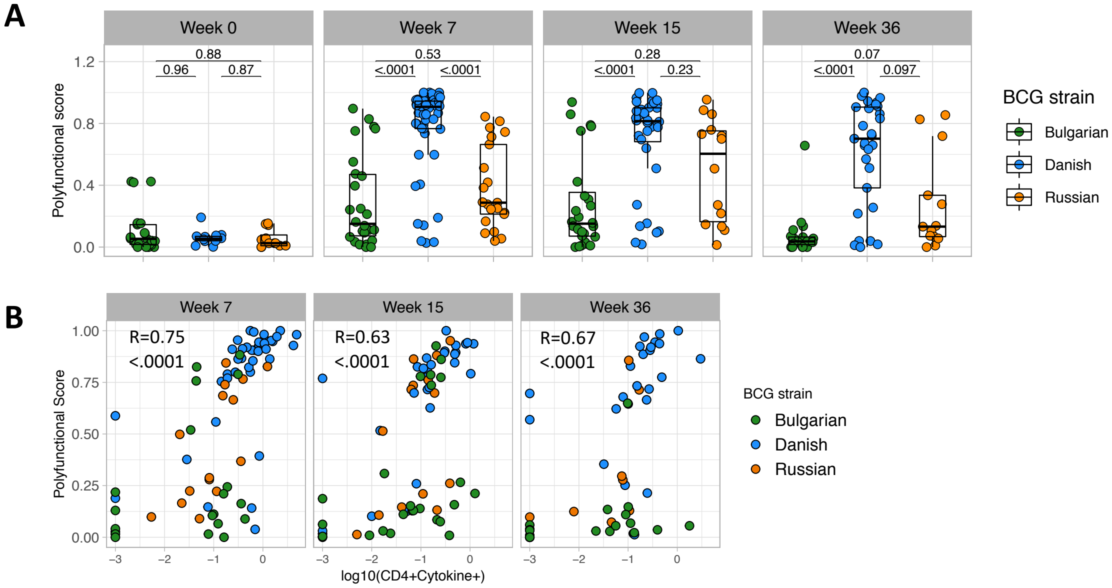

Supplementary 3 Polyfunctionality scores for BCG responses

(A) Cross-sectional analysis of Polyfunctional scores (as calculated in COMPASS model) stratified by BCG immunizing strain [Green (BCG-Bulgaria), blue (BCG-Denmark), orange (BCG-Russia)]. **(B)** Correlation between Polyfunctional scores and cytokine response magnitude. Spearman's ranked correlation coefficient is reported for N=109 infants in all BCG strain groups. Mann Whitney U test was used to test for differences by BCG strain. Adjusted P-values are reported with $P<.05$ were considered significant after multiple comparisons correction.

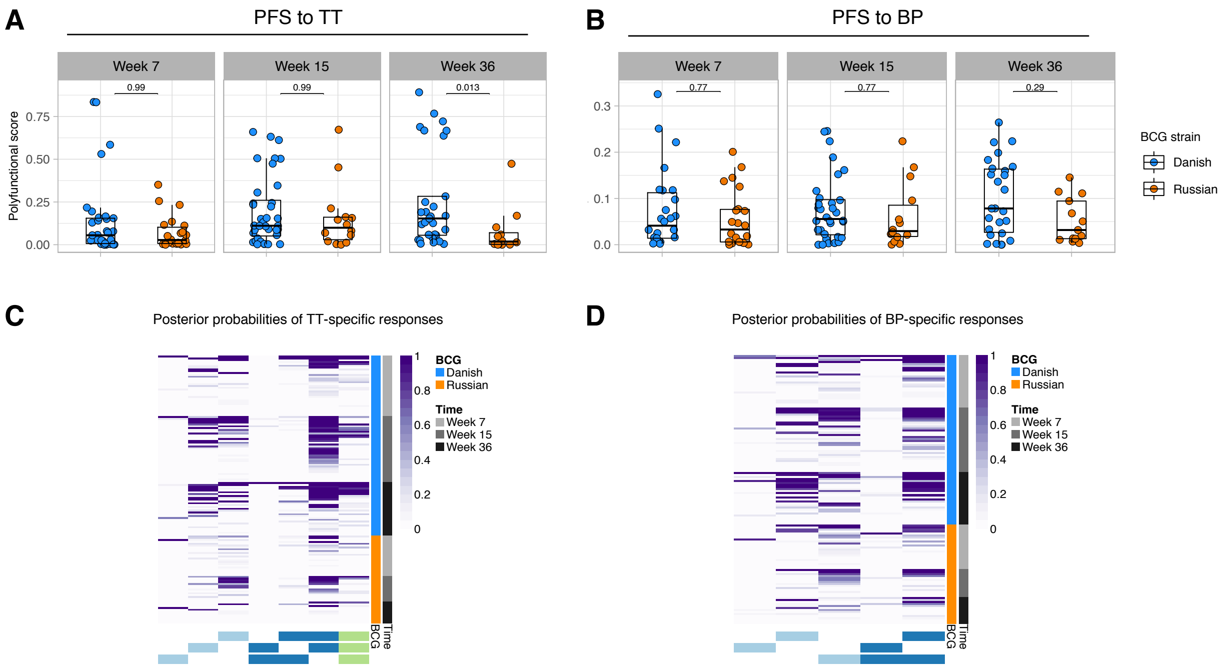

Supplementary 4 Polyfunctionality scores for Tetanus and Pertussis responses
(A&B) Jitter plots show cross-sectional Polyfunctional scores (PFS) to TT and BP antigens (as calculated in COMPASS model) stratified by BCG immunizing strain. Mann Whitney U test was used to test for differences by BCG strain. Adjusted P-values are reported with P<.05 were considered significant after multiple comparisons correction. **(C&D)** Heatmap of posterior probabilities of TT and BP responses estimated by COMPASS analysis (annotations are as described in Figure 2D).
